# Development and validation of a novel high-performance liquid chromatography (HPLC) method for the detection of related substances of pralsetinib, a new anti-lung cancer drug

**DOI:** 10.3389/fchem.2024.1450692

**Published:** 2024-08-21

**Authors:** Yonghong Zhu, Jisu Qin, Wenyi Wu, Liangliang Cai

**Affiliations:** ^1^ Department of Pharmacy, Affiliated Nantong Hospital of Shanghai University (The Sixth People’s Hospital of Nantong), Nantong, Jiangsu, China; ^2^ Department of Pharmacy, Affiliated Hospital of Nantong University, Pharmacy School of Nantong University, Nantong, China; ^3^ Department of Quality Inspection, Sinopharm Holding Nantong Ltd., Nantong, China

**Keywords:** pralsetinib, liquid chromatography, method development, method validation, related substances

## Abstract

**Background:**

Pralsetinib, a targeted inhibitor of the RET enzyme, plays a critical role in the treatment of adult patients with locally advanced or metastatic non-small cell lung cancer (NSCLC) characterized by RET gene fusion mutations following platinum-based chemotherapy. Nevertheless, impurities resulting from the manufacturing and degradation of pralsetinib have the potential to impact its therapeutic effectiveness and safety profile.

**Methods:**

To address this issue, a liquid chromatography method was developed and validated for the specific identification of pralsetinib and its related impurities. The separation of pralsetinib and its related impurities was achieved via a Waters X Bridge C_18_ column with dimensions of 4.6 mm × 250 mm and a particle size of 5 μm. Mobile phase A was composed of 20 mmol/L potassium dihydrogen phosphate (KH_2_PO4) and acetonitrile (ACN) at a volume ratio of 19:1, while mobile phase B consisted solely of ACN, utilizing a gradient elution technique. Detection was performed at a wavelength of 260 nm, with an injection volume of 10 μL and a flow rate of 1.0 mL/min.

**Results:**

The chromatographic method established in this study was validated according to the ICH Q2 (R1) guidelines. The method demonstrated excellent linearity over a specific concentration range (imp-A: 0.035–10.21 μg/mL; imp-B: 0.09–10.16 μg/mL; imp-C: 0.15–10.19 μg/mL; pralsetinib: 0.04–10.32 μg/mL). Additionally, the method possesses high sensitivity, with detection limits for impurities A, B, C, and pralsetinib of 0.01, 0.03, 0.015, and 0.013 μg/mL, respectively, and quantification limits of 0.035, 0.09, 0.05, and 0.04 μg/mL, respectively. In terms of specificity, stability, repeatability, accuracy, and robustness, the method met the validation acceptance criteria. Overall, the chromatographic technique established in this study can effectively separate pralsetinib and its impurities, providing reliable assurance for the accurate detection and quantification of impurities.

**Conclusion:**

The chromatographic method developed in this study can be utilized for the detection of pralsetinib and its impurities, offering a crucial reference for research on the quality of pralsetinib.

## 1 Introduction

Non-small cell lung cancer (NSCLC) is the most common type of lung cancer, accounting for approximately 85%–90% of all lung cancer cases (Chen et al., 2022; Melosky et al., 2022). Unlike other types of lung cancer, NSCLC usually does not exhibit symptoms in the early stages, which leads to delayed diagnosis and often results in its discovery in the later stages (Du et al., 2022; Spagnuolo and Gridelli, 2023). To improve the treatment outcomes of NSCLC, scientists have conducted extensive research aiming to identify specific therapeutic targets for interventional treatment. Currently, the clinically targeted therapeutic drugs for non-small cell lung cancer mainly include epidermal growth factor receptor (EGFR) inhibitors (Yu et al., 2020; Cheng et al., 2021), kirsten rat sarcoma viral oncogene homologue (KRAS) inhibitors (Sidaway, 2021; Nakajima et al., 2022; Ou et al., 2022), anaplastic lymphoma kinase (ALK) inhibitors (Frampton, 2013; Camidge et al., 2019; Shaw et al., 2020), and (rearranged during transfection) RET inhibitors (Huang et al., 2023; Zhou et al., 2023).

RET is a transmembrane glycoprotein receptor tyrosine kinase encoded by the RET proto-oncogene, which is located on chromosome 10q11.2 and plays a pivotal role in the embryonic development of the kidney and enteric nervous system (Yadav et al., 2020; Buchholz et al., 2021). The presence of RET fusion, an oncogenic driver, is observed in approximately 1%–2% of individuals with NSCLC (Lin et al., 2020). Targeted therapies directed at RET have demonstrated notable efficacy in NSCLC, leading to enhanced response rates and extended disease-free survival for patients.

Pralsetinib, chemically known as (((R)-3-(6-(4-fluoro-1H-pyrazol-1-yl)pyridin-3-yl)-1-((1s,4S)-1-methoxy-4-(4-methyl-6-((5-methyl-1H-pyrazol-3-yl)amino)pyrimidin-2-yl) cyclohexyl) butan-1-one--(S)-1-(6-(4-fluoro-1H-pyrazol-1-yl)pyridin-3-yl) ethan-1-amine), a highly selective RET inhibitor (Griesinger et al., 2022), was approved by the China National Medical Products Administration in March 2021 for the treatment of locally advanced or metastatic non-small cell lung cancer in adult patients with RET gene fusion who previously received platinum-based chemotherapy.

Current research on pralsetinib primarily focuses on its clinical effectiveness and safety in treating NSCLC (Gainor et al., 2021; Griesinger et al., 2022). Meanwhile, studies have employed HPLC-MS/MS technology to detect drug concentrations in the plasma of patients taking pralsetinib (Gulikers et al., 2023). However, it is noteworthy that no literature has yet reported the use of high-performance liquid chromatography (HPLC) to detect related substances in pralsetinib’s bulk drug. The process impurities and degradation impurities that may be generated during the synthesis and storage of pralsetinib may affect its safety and efficacy. Therefore, developing a detection method for pralsetinib and its impurities.

The high-performance liquid chromatography (HPLC) technique, characterised by its convenience, simplicity, stability, and cost-effectiveness, remains the most ideal separation technique for determining active ingredients and related substances in pharmaceutical samples in the pharmaceutical industry (Zhao et al., 2022). Furthermore, the majority of bulk medications included in the United States Pharmacopeia (USP) and the European Pharmacopeia (EP) utilize HPLC to detect related substances (Agtas et al., 2024; Gondhale-Karpe and Manwatkar, 2023; Zhao and Rustum, 2024). Currently, there is a scarcity of documented literature on techniques for detecting related substances in pralsetinib’s bulk drug. Considering the potential impact of impurities on pralsetinib’s efficacy and safety, it is crucial to develop an HPLC method for detecting relevant substances in pralsetinib’s bulk drug.

This research presents the successful development and implementation of a RP-HPLC method for detecting impurities in pralsetinib. This method is distinguished by its simplicity, sensitivity, accuracy, and durability. In particular, this approach can efficiently separate pralsetinib-related substances, including some unknown impurities and known impurities. Furthermore, the specificity, accuracy, stability, and robustness of the method were assessed. The RP-HPLC method was assessed for its limit of quantitation (LOQ), limit of detection (LOD), linearity, and recovery rate simultaneously. In conclusion, the established RP-HPLC method offers a novel approach for the advancement of process development and quality assessment of pralsetinib.

## 2 Materials and methods

### 2.1 Chemicals and reagents

Pralsetinib and its known impurities (named imp-A, imp-B, and imp-C) were acquired from Beijing Bailingwei Technology Co., Ltd. (Beijing, China). Merck (Darmstadt, Germany) provided HPLC-grade ACN and methanol (MeOH). Other analytical grade chemical reagents were purchased from China National Pharmaceutical Group Chemical Reagent Co., Ltd. (Beijing, China).

### 2.2 Instruments

An Agilent 1200 HPLC system, which was outfitted with an ultraviolet (UV) detector, and a Shimadzu LC-20AD system, which was equipped with a photodiode array detector, were used for method development and validation.

### 2.3 HPLC conditions

A chromatographic column (4.6 mm × 250 mm, 5 μm) was used to separate pralsetinib and its impurities at a flow rate of 1.0 mL/min. Mobile phase A is composed of a 20 mmol/L aqueous solution of potassium dihydrogen phosphate (KH_2_PO_4_) and acetonitrile (ACN) at a volume ratio of 19:1, whereas mobile phase B is composed solely of acetonitrile (ACN). Table 1 presents a thorough explanation of the gradient elution procedure. UV analysis was conducted at a wavelength of 260 nm, with an injection volume of 10 µL.

**TABLE 1:** Gradient program of the final method.

Time (min)	Mobile phase A (%)	Mobile phase B (%)
0	92	8
2	92	8
18	62	38
28	60	40
35	40	60
40	40	60
41	92	8
50	92	8

### 2.4 Preparation of stock solutions

#### 2.4.1 Preparation of pralsetinib stock solutions

Approximately 25 mg of pralsetinib was precisely weighed and transferred to a 50 mL volumetric flask. Next, a suitable volume of 50% methanol-water mixture was added, and the mixture was sonicated for dissolution. The volume was subsequently adjusted to the mark value to obtain the pralsetinib stock solution.

#### 2.4.2 Preparation of pralsetinib-related substance stock solutions

The impurities (imp-A, imp-B, and imp-C) were measured at approximately 10 mg each and transferred individually to 20 mL volumetric flasks. The impurities were subsequently dissolved in a 50% methanol solution using ultrasonication and diluted to the calibration mark, yielding a stock solution of impurities with a concentration of 0.5 mg/mL.

### 2.5 Preparation of mixed solutions and system suitability solutions

A total of 10 mg of pralsetinib was accurately weighed and transferred to 20 mL volumetric flask. The compounds were subsequently dissolved in 10 mL of methanol, and 5 mL of a 1 mol/L hydrochloric acid (HCl) solution was added. The resulting mixture was heated at 75°C for 48 h. Upon completion of the reaction, the mixture was neutralised with a 1 mol/L sodium hydroxide (NaOH) solution. Next, the mixture was diluted with a 50% methanol aqueous solution to a total volume of 20 mL to obtain the acidic degradation product.

In a comparable manner, 10 mg of pralsetinib should be accurately weighed and transferred into a 20 mL volumetric flask, followed by the addition of 10 mL of methanol for sonication to facilitate dissolution. Subsequently, 10 mL of a 3% hydrogen peroxide solution was added, and the reaction was allowed to proceed at ambient temperature for a duration of 1.5 h. Following the completion of the reaction, it was suggested to that the reaction be terminated with manganese dioxide, followed by filtration to separate the manganese dioxide and collect the resulting solution containing the oxidative degradation product. Finally, the acidic degradation product and the oxidative degradation product should be combined at a ratio of 3:1 to generate a system suitable solution.

### 2.6 Preparation of the sample solution

For sample solution preparation, approximately 10 mg of pralsetinib bulk drug were precisely measured and then dissolved in a mixture of methanol and water mixture (50/50, volume/volume) until a concentration of approximately 0.5 mg per milliliter was reached.

## 3 Results and discussion

### 3.1 Method development

**FIGURE 1:**
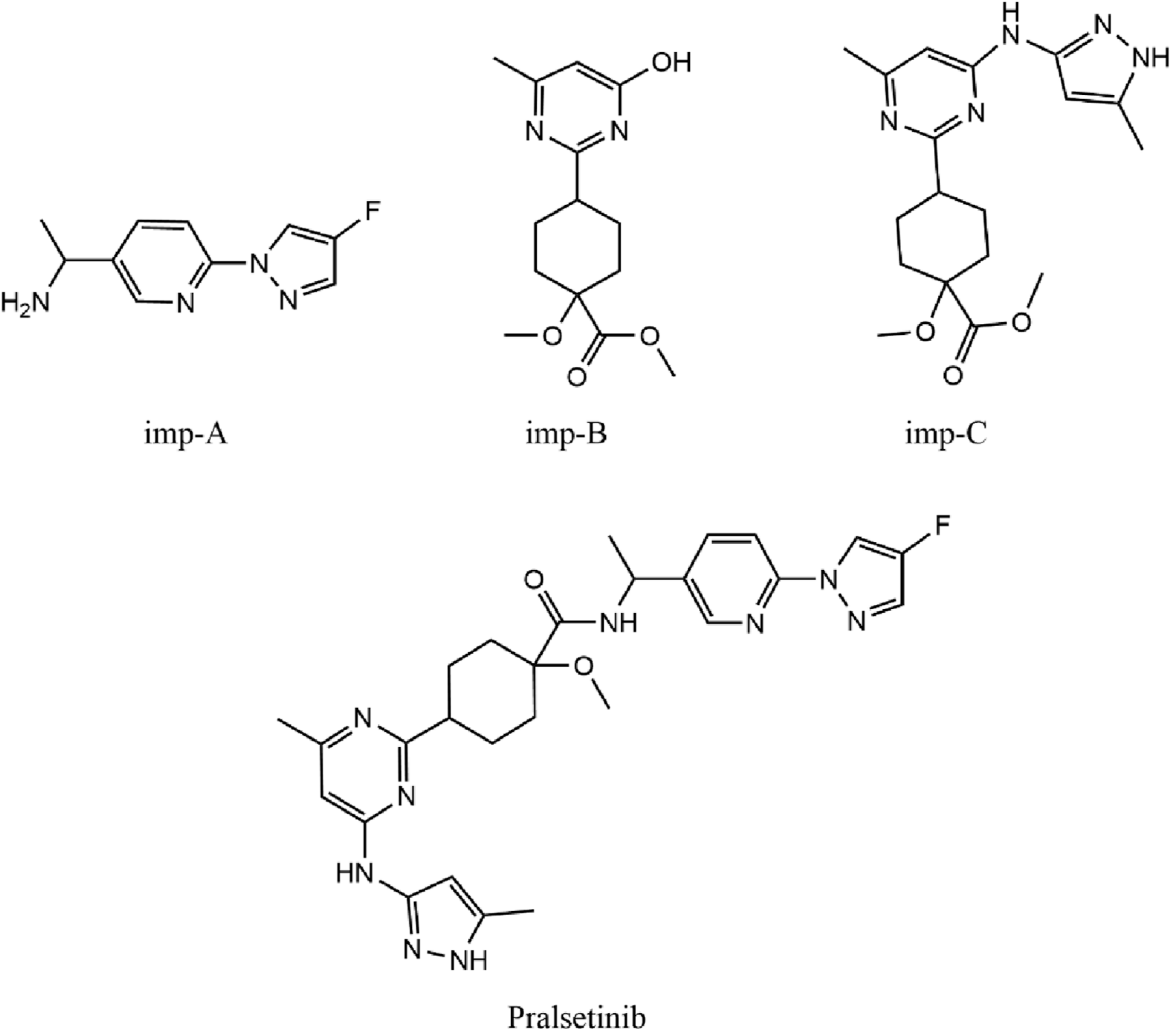
Chemical structures of pralsetinib, imp-A, imp-B and imp-C.

The synthesis of the RET inhibitor pralsetinib was conducted according to the synthetic pathway outlined in Supplementary Figure S1 of the US patent US20170121312A1. The synthesis of pralsetinib consists of seven steps and uses various reagents and intermediates. Figure 1 depicts the chemical structures of three known impurities and pralsetinib studied. Imp-B and imp-C are impurities not only from photodegradation, but also from the manufacturing process.

The current preliminary research on pralsetinib primarily encompasses two significant aspects: evaluating the efficacy and safety of pralsetinib in treating NSCLC, as well as monitoring and analyzing its serum drug concentration. Notably, no studies have been reported thus far regarding the detection of pralsetinib-related substances.

**FIGURE 2:**
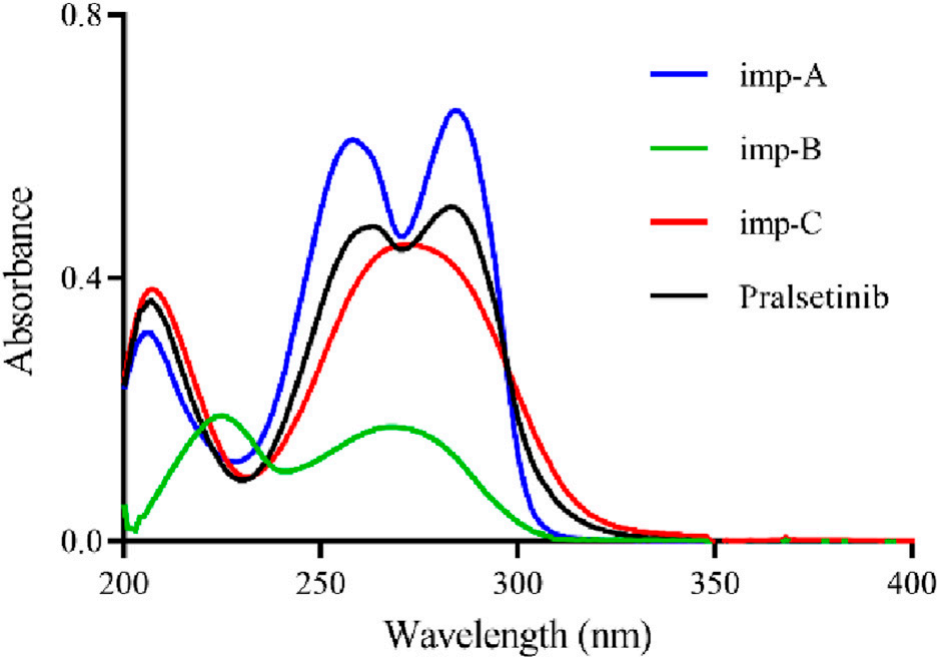
UV spectra of pralsetinib and its known impurities (imp-A, imp-B, imp-C).

To optimise the detection method, an investigation was conducted on the impact of the solvent, detection wavelength, mobile phase composition, and elution method on sample separation. The selection of an appropriate solvent involved dissolving pralsetinib in both methanol (MeOH) and acetonitrile (ACN). The results of the experiments indicated that pralsetinib was less soluble in acetonitrile than in methanol, which completely dissolved the sample. Owing to the volatile nature of methanol, a 50% methanol-water mixture was also tested as a solvent, revealing that the sample could still be fully dissolved at a concentration of 0.5 mg/mL. Following the solution stability study, it was determined that there was no notable alteration in related substances within the sample when a 50% methanol-water mixture was used as the solvent within a period of 48 h. Consequently, the selected solvent for this investigation was ultimately a 50% methanol-water mixture. To identify the most suitable detection wavelength, pralsetinib and its known impurities (imp-A, imp-B, imp-C) were diluted with a 50% methanol-water mixture to a concentration of 10 μg/mL and subjected to UV-VIS scanning analysis within the wavelength range of 200–400 nm. The UV spectrum, depicted in Figure 2, revealed prominent absorption peaks near 260 nm for both pralsetinib and its impurities. Consequently, the detection wavelength for this study was 260 nm.

**FIGURE 3:**
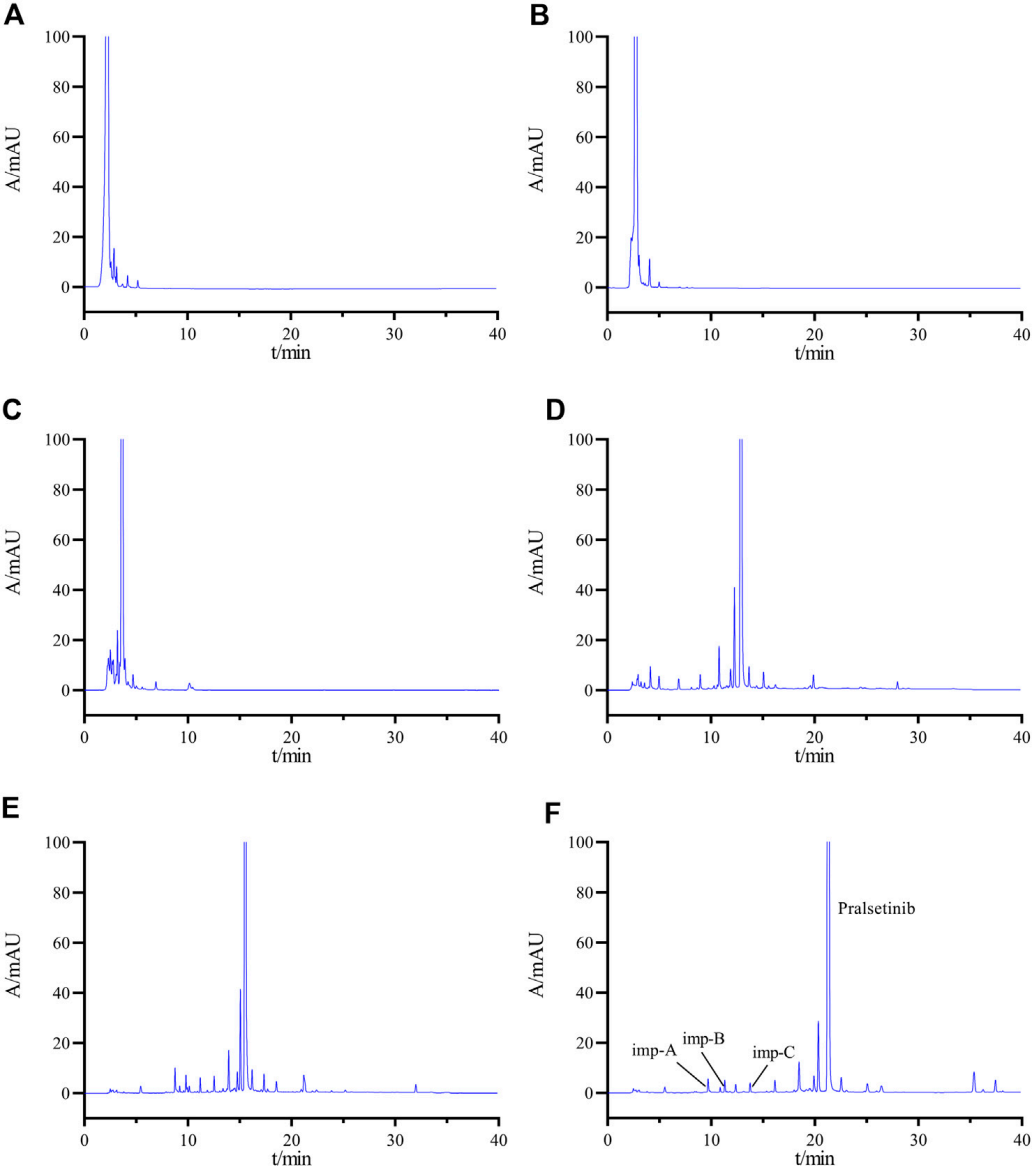
Chromatogram obtained by optimizing the HPLC conditions **(A)** ACN-H_2_O (70:30); **(B)** ACN-20 mM KH_2_PO4 solution (70:30); **(C)** ACN-20 mM KH_2_PO4 solution (50:50); **(D)** gradient elution Condition 1; **(E)** gradient elution Condition 2; **(F)** final determined gradient elution condition.

When developing the analytical method, the system suitability solution was selected because of the presence of a large number of impurities and difficult-to-separate chromatographic peak pairs. The peak shape, retention time, and peak resolution was used as evaluation criteria. During the development of the analytical methodology, the system suitability solution was chosen because of its complexity, containing numerous impurities and challenging chromatographic peak pairs. The evaluation process emphasized three key metrics: peak symmetry, retention time, and peak resolution. We preferred to utilize high-performance liquid chromatography (HPLC) with an isocratic elution strategy, emphasizing the simplicity of the mobile phase composition. The inclusion of buffer salts was minimized when possible. Our initial experiments involved the use of a mobile phase ratio of ACN-H_2_O (70:30, v/v). Despite the primary peak having a retention time of 2.271 min, overlap among impurity peaks was observed, leading to a compromise in separation efficiency, as illustrated in Figure 3A. Owing to the weak basic characteristics of pralsetinib, the incorporation of buffer salts to improve chromatographic separation was explored. Specifically, we utilized acetonitrile and 20 mM potassium dihydrogen phosphate (KH_2_PO_4_) as mobile phase components, and evaluated two ratios: 70:30 and 50:50 (v/v). However, the experimental results (depicted in Figures 3B, C) revealed limited improvement in peak resolution between the main peak and impurities.

Subsequently, an investigation was conducted on the impact of gradient elution. The established gradient elution condition 1 (0–2 min, 20% B →20% B; 2–20 min, 20% B → 70% B; 20–30 min, 70% B → 70% B; 30–31 min, 70% B → 20% B; 31–40 min, 20% B → 20% B) revealed a minimum separation of 1.08 (<1.2) between adjacent impurity peaks and a separation of 3.96 between the main peak and the impurity peaks (Figure 3D), failing to meet the specified criteria. Therefore, the initial organic phase ratio of the gradient was reduced, and the gradient change rate was adjusted to establish gradient elution condition 2. (0–2 min, 8% B→8% B; 2–20 min, 8% B →70% B; 20–30 min, 70% B→70% B; 30–31 min, 70% B→8% B; 31–40 min, 8% B →8% B). Under these conditions, the separations between the main peak and adjacent impurities and between impurities improved, but still did not meet the experimental requirements (Figure 3E). After multiple adjustments, we finally determined the gradient elution conditions as described in Section 2.3 (Figure 3F).

### 3.2 Method validation

Validation of the HPLC method for detecting related substances in the receptor tyrosine kinase inhibitor pralsetinib was carried out as per the guidelines set by the International Council for Harmonization of Technical Requirements for Pharmaceuticals for Human Use (ICH) Guideline Q2 (R1) (Guideline, 2005). This validation encompasses specificity, sensitivity, solution stability, linearity, precision, accuracy, and robustness.

#### 3.2.1 Specificity

**FIGURE 4:**
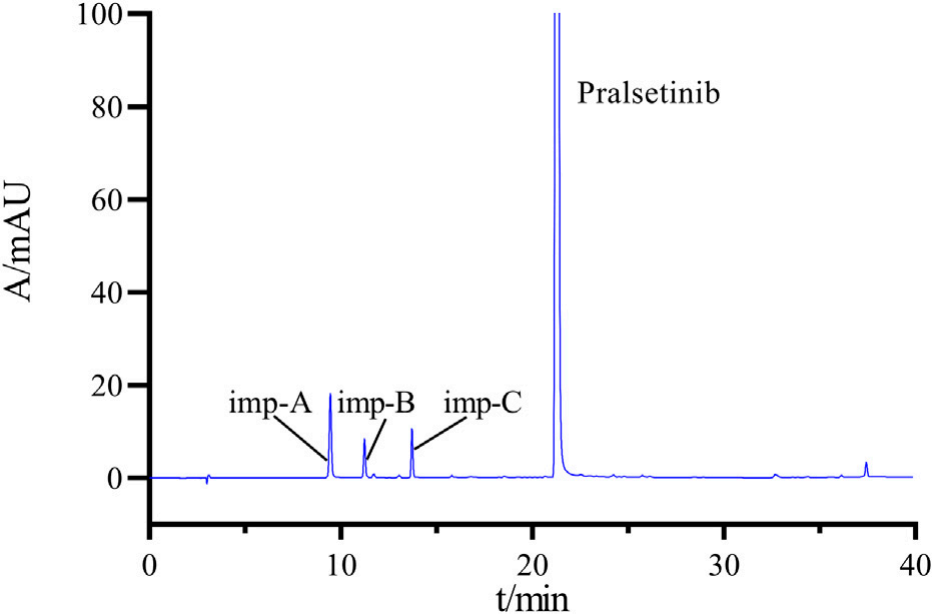
Chromatograms of mixed impurity solutions.

Following the chromatographic parameters outlined in Section 2.3, the solvent, mixed impurities, and system suitability solution were injected to assess the specificity of the detection method. Figure 4 shows the chromatogram of the mixed impurity solution, where Peaks one to four are identified as imp-A, imp-B, imp-C, and pralsetinib, respectively. The retention time (RT), relative retention time (RRT), and resolution values can be found in Supplementary Table S1. The minimum resolution between these impurities and pralsetinib is calculated to be 4.98 (>1.5), satisfying the experimental requirements.

Additionally, the chromatogram of the system suitability solution is depicted in Figure 3F, revealing resolutions of 5.01 and 6.15 between the main peak and adjacent impurity peaks, surpassing the threshold of 1.5. Moreover, the minimum resolution between impurity peaks is 1.49, exceeding the threshold of 1.2. Collectively, these findings indicate that the method displays favorable specificity.

#### 3.2.2 Forced degradation experiments

For the forced degradation study, approximately 10 mg of pralsetinib was weighed and transferred to a 20 mL volumetric flask. The compound was then dissolved in methanol and subjected to a range of degradation conditions, such as acid and alkali hydrolysis, oxidation, photolysis, and heat degradation.

To simulate acid-induced stress, the pralsetinib solution was subjected to treatment with 1 mol/L HCl at a temperature of 75°C for 48 h. The process of alkaline degradation involved the use of a 1 mol/L NaOH solution at a temperature of 75°C for 72 h, followed by the application of a 10% H_2_O_2_ solution for 1.5 h. These conditions were used to mimic the degradation effects of alkali and oxidation. The pralsetinib sample was subjected to thermal degradation at 100°C for a period of 30 days, and photodegradation was induced by exposure to an LED tube with an intensity of 4,500 lx for a duration of 30 days. The sample was subsequently diluted to 0.5 mg/mL via a mixture of methanol and water (v/v 1:1) and analyzed according to the procedure specified in Section 2.3.

**FIGURE 5:**
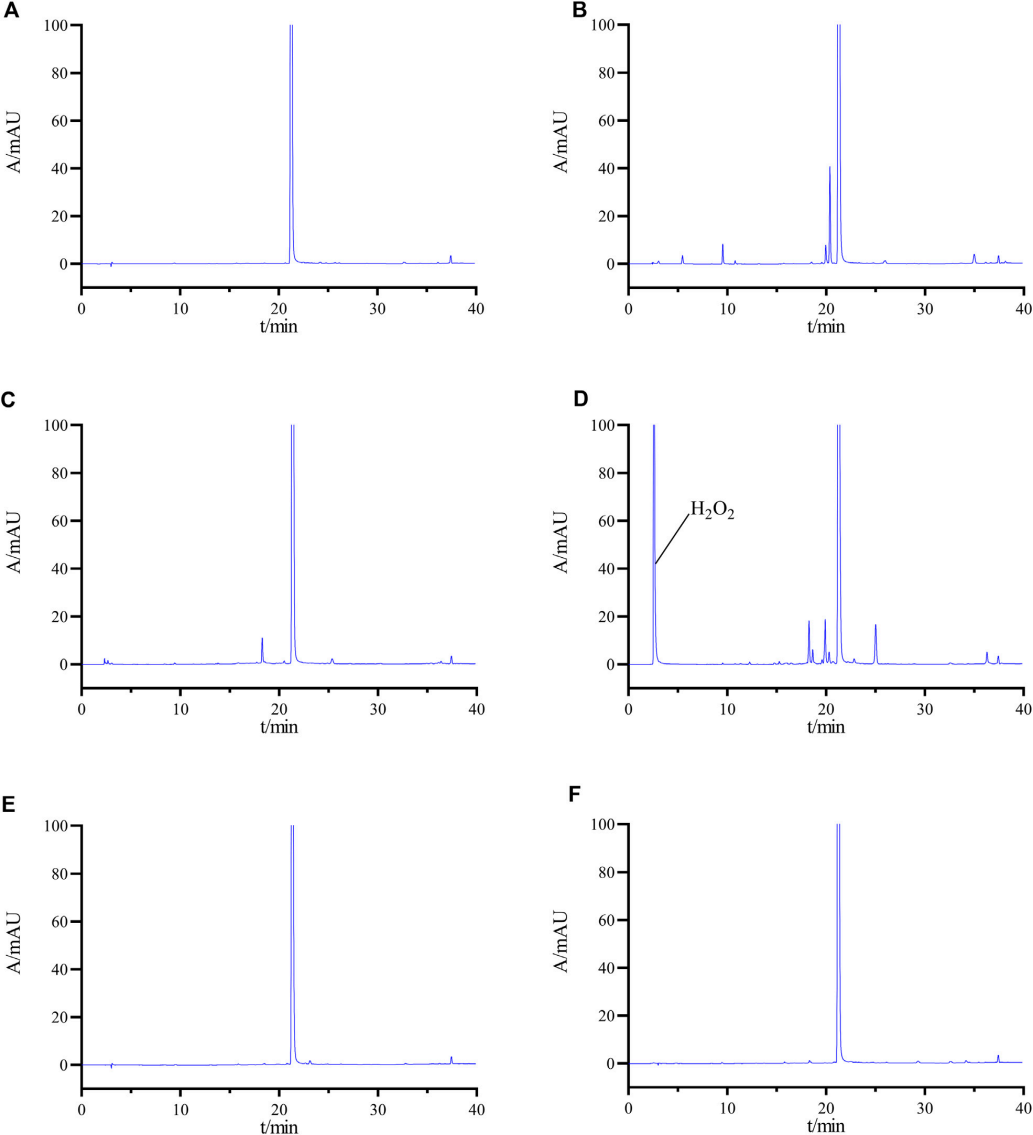
HPLC chromatograms of undegraded **(A)**, acid-degraded **(B)**, base-degraded **(C)**, oxidatively degraded **(D)**, heat-degraded **(E)**, and photolytically degraded **(F)** samples.

Figure 5 shows the chromatographic results obtained from the forced degradation study of pralsetinib. Supplementary Table S2 presents an evaluation of the compound’s stability when subjected to different forced degradation conditions, considering the number of impurities, main peak content, minimum separation between principal components and impurities, minimum separation between impurities, and equilibrium rate. Pralsetinib is notably stable under conditions of elevated temperature, exposure to light, and alkaline environments, although it is susceptible to degradation under acidic and oxidative conditions. Importantly, despite differing degradation conditions, the minimum resolution difference between the main peak and impurities exceeds 1.5, whereas the minimum resolution difference among impurities surpasses 1.2, thereby meeting the specified criteria. Additionally, the equilibrium rate falls within the range of 95%–105%. This range is commonly accepted as a critical criterion for achieving equilibrium in material balance.

#### 3.2.3 LODs and LOQs

The sensitivity of the detection method was assessed by determining the limit of detection (LOD) and limit of quantification (LOQ) through stepwise dilution of stock solutions of pralsetinib and its identified impurities (imp-A, imp-B, imp-C), followed by computation of signal-to-noise ratio. The concentrations corresponding to signal-to-noise (S/N) ratios of 3:1 and 10:1 were designated the LOD and LOQ, respectively. The detailed results can be found in Table 2.

**TABLE 2:** Linearity, LODs and LOQs of pralsetinib and known impurities (imp-A, imp-B, imp-C).

Substance	Standard calibration curves	Correlation coefficient (r)	LOD (μg/mL)	LOQ (μg/mL)
Pralsetinib	y = 22.04x + 2.767	0.9991	0.013	0.04
imp-A	y = 27.37x ‒ 1.107	0.9989	0.010	0.035
imp-B	y = 10.86x ‒ 1.204	0.9993	0.030	0.09
imp-C	y = 11.64x ‒ 1.536	0.9990	0.015	0.05

#### 3.2.4 Stability of the solution

A thorough stability assessment was conducted on the pralsetinib sample solution by analyzing it at different time points (0, 1, 2, 4, 6, 8, 12, and 24 h) to determine its stability. This assessment included monitoring changes in the number of impurities, maximum content of individual impurities, and overall impurity content. The results of these analyses are detailed in Supplementary Table S3. Additionally, after being exposed to room temperature for 24 h, the sample mixture presented minimal alterations in the three parameters, indicating its stability at room temperature for a period of 24 h.

To evaluate the stability of solutions containing known impurities, a mixed impurity solution was prepared and analysed via injection at various time points over a 24-h period using the chromatographic parameters specified in Section 2.3. The results indicated that the relative standard deviations of the peak areas for pralsetinib and impurities A, B, and C were 1.35%, 1.27%, 1.39% and 1.73%, respectively, which are all below the predetermined 2% threshold. These findings suggest that solutions containing these impurities remain stable for 24 h.

#### 3.2.5 Linearity

This study presents an investigation of the linear relationship between pralsetinib and its related substances at concentrations ranging from the LOD to 2.0% of the desired concentration (approximately 0.5 mg/mL).

To conduct the analysis, multiple concentrations of detection solutions were prepared by diluting the prasetinib standard stock solution and the impurity (imp-A, -B, and -C) standard stock solutions with a MeOH/H_2_O mixture (50/50, v/v). The linearity of the method was assessed through chromatographic analysis. The regression curve for pralsetinib and its related substances is shown in Supplementary Figure S2, while Table 2 presents the calibration curve and correlation coefficient. Across the analytical concentration range of the LOD to 2.0% of the target concentration (∼0.5 mg/mL), all correlation coefficients surpassed 0.99.

#### 3.2.6 Precision

The precision of the instrument was assessed by performing six consecutive injections of the mixed solution described in Item 2.5 under the specific HPLC conditions outlined in Item 2.3. Chromatographs were subsequently generated to quantify both the relative retention time and peak area. The relative standard deviations (RSDs) for pralsetinib and its impurities (imp-A, -B, and -C) were found to be 0.04%, 0.06%, and 0.08%, respectively. Similarly, the RSDs for the peak areas were determined to be 0.85%, 0.65%, 1.21% and 0.75%, respectively. Notably, all RSD values were less than 2%, indicating a high level of precision in the instrument.

#### 3.2.7 Repeatability

To evaluate the reproducibility of the method, six samples with known impurities were prepared and subsequently analysed under the conditions outlined in Section 2.3. The RSD for impurities A, B, and C were calculated to be 0.57%, 0.79%, and 1.32%, respectively. These findings, which all fall below the 2% threshold, suggest that the method has satisfactory reproducibility.

#### 3.2.8 Recovery

This study tested the recovery rates of known impurities (imp-A, imp-B, and imp-C) from pralsetinib at three different concentrations, 50%, 100%, and 150%, with a concentration of approximately 1 μg/mL used as the reference concentration at the 100% level. To achieve these concentrations, different volumes of impurity stock solutions were added to the pralsetinib sample solution. Each concentration of the sample was prepared in triplicate and injected, and the recovery rate was calculated. The findings depicted in Table 3 indicate that the recoveries of all known impurities ranged from 50% to 150%, with RSD values for these compounds below 2.0%. These findings suggest that the method demonstrates a high level of accuracy.

**TABLE 3:** Recovery of known impurities (imp-A, imp-B, imp-C) in pralsetinib.

Substance	Target Level (%)	Spiked Conc. (μg/mL)	Determined Conc. (μg/mL)	Recovery (%)	Average recovery (%)	RSD (%)
imp-A	50	0.511	0.502	98.33	101.01	1.56
		0.511	0.513	100.49		
		0.511	0.527	103.23		
	100	1.021	1.042	102.06		
		1.021	1.028	100.69		
		1.021	1.035	101.37		
	150	1.532	1.542	100.69		
		1.532	1.575	102.84		
		1.532	1.522	99.38		
imp-B	50	0.508	0.501	98.62	99.03	1.89
		0.508	0.492	96.85		
		0.508	0.497	97.83		
	100	1.016	1.008	99.21		
		1.016	1.002	98.62		
		1.016	0.997	98.13		
	150	1.524	1.535	100.72		
		1.524	1.496	98.16		
		1.524	1.572	103.15		
imp-C	50	0.510	0.489	95.98	99.13	1.78
		0.510	0.495	97.15		
		0.510	0.503	98.72		
	100	1.019	1.035	101.57		
		1.019	1.025	100.59		
		1.019	1.009	99.02		
	150	1.529	1.539	100.69		
		1.529	1.512	98.92		
		1.529	1.522	99.57		

#### 3.2.9 Durability

To assess the durability of the method, we determined the optimal parameters for the system under various chromatographic conditions. These conditions included variations in the initial ratio of mobile phases A-B, wavelength, column temperature, flow rate, and column type. A comprehensive overview of the conditions for durability can be found in Table 4.

**TABLE 4:** Test conditions of robustness.

Chromatogram conditions	The variation range of parameters
The initial proportion of mobile phases A-B (%)	94:6; 92:8; 90:10
Wavelength (nm)	255, 260, 265
Column temperature (°C)	35, 40, 45
Flow rate (mL/min)	0.9, 1.0, 1.1
Chromatographic column	Waters-C_18,_ Agilent-5HC-C_18,_ Luna-C_18_

The resolution between pralatinib and its adjacent impurity peaks was greater than 1.5 under various acceptable conditions, with the minimum resolution between impurity peaks exceeding 1.2. There were no notable changes observed in the quantity and composition of impurities. Furthermore, adjustments to the column temperature and wavelength did not affect the detection of related substances. However, variations in the flow rate, column type, and initial mobile phase ratio had minimal effects on the retention time and resolution. Fortunately, minor modifications to these parameters did not substantially impact the detection results, effectively highlighting the robustness of the methodology.

#### 3.2.10 Sample detection

Accurately weigh 10 mg of pralsetinib’s bulk drug from each of the three different batches and place them individually in 20 mL volumetric flasks. Subsequently, dissolve the samples using a 50% methanol-water solution and dilute to the volumetric flask’s mark. Afterward, perform the detection according to the chromatographic conditions outlined in “Item 2.3.” The results of the detection are presented in Table 5.

**TABLE 5:** Results of related substances in three batches of pralsetinib samples.

Batch number	Related substances (%)				
	imp-A	imp-B	imp-C	Other single maximum impurity	Total impurities
20230821	0.08	0.05	0.09	0.09	0.38
20231215	0.06	0.07	0.10	0.13	0.49
20240301	0.11	0.06	0.07	0.11	0.42

## 4 Conclusion

This study aimed to develop an analytical method for detecting process impurities generated during the synthesis of pralsetinib and degradation impurities resulting from forced degradation experiments. Based on the characteristics of these impurities, a new RP-HPLC-UV method was developed for the quantitative analysis of pralsetinib and its related substances. The method involved evaluating factors critical to separation efficiency, including solvents, detection wavelength, mobile phase composition, elution methods, and chromatographic columns. The method was validated in accordance with ICH guidelines, exhibiting satisfactory sensitivity, specificity, accuracy, linearity, repeatability, and robustness, thereby meeting the criteria for method validation. Consequently, this research offers valuable insights for the quality assurance of pralsetinib.

## Data Availability

The original contributions presented in the study are included in the article/Supplementary Material, further inquiries can be directed to the corresponding author.
