# Supplementary material for: Development and validation of a novel high-performance liquid chromatography (HPLC) method for the detection of related substances of pralsetinib, a new anti-lung cancer drug
Source: Front Chem. 2024 Aug 21;12:1450692. doi: 10.3389/fchem.2024.1450692 (PMC11371568; doi:10.3389/fchem.2024.1450692)
Supplement: Supplementary file 1 [file DataSheet1.docx]

**Supplementary Material**

**Development and validation of a novel high-performance liquid chromatography (HPLC) method for the detection of related substances of pralsetinib, a new anti-lung cancer drug**


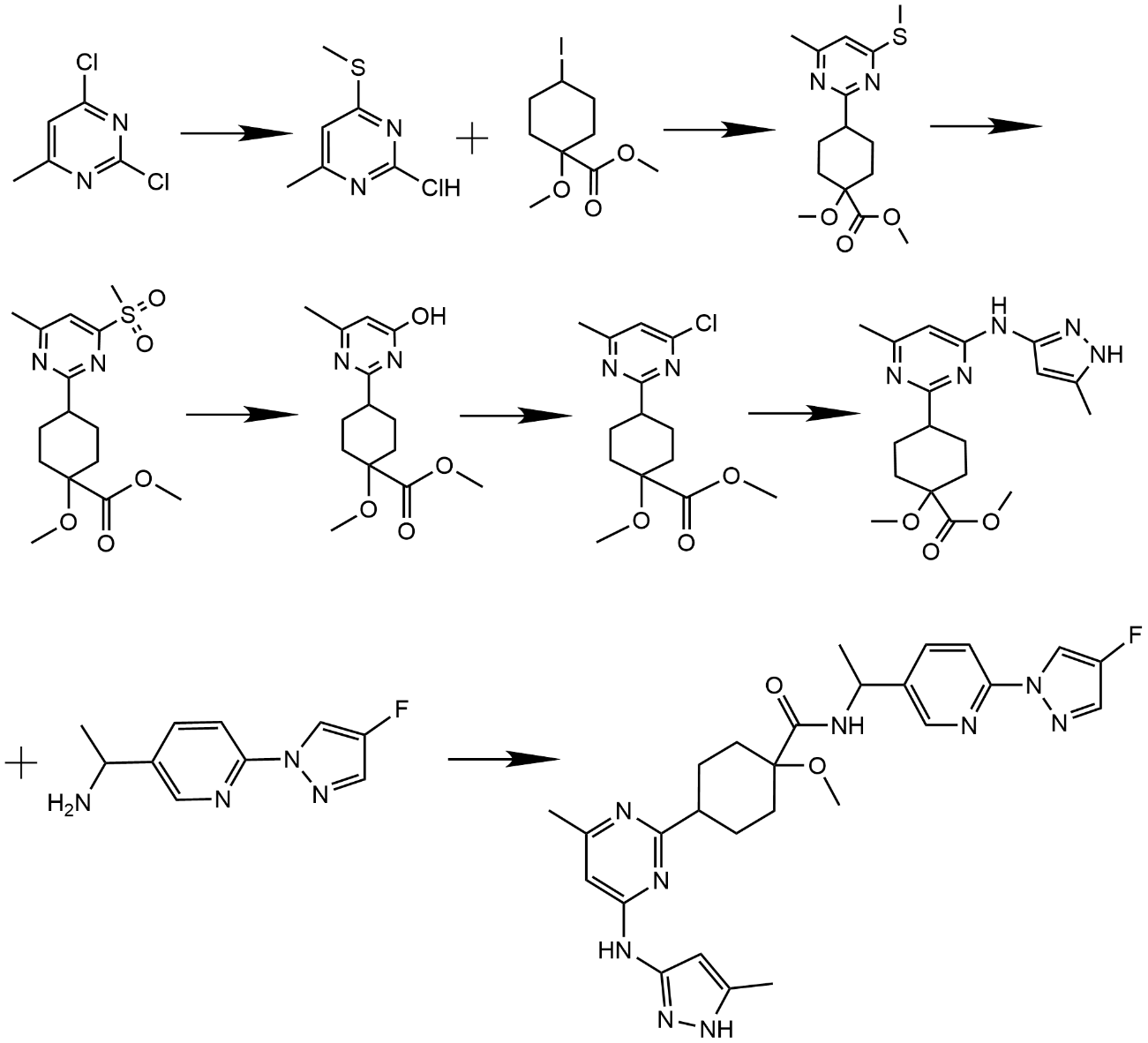


**Figure S1** Synthetic route of pralsetinib


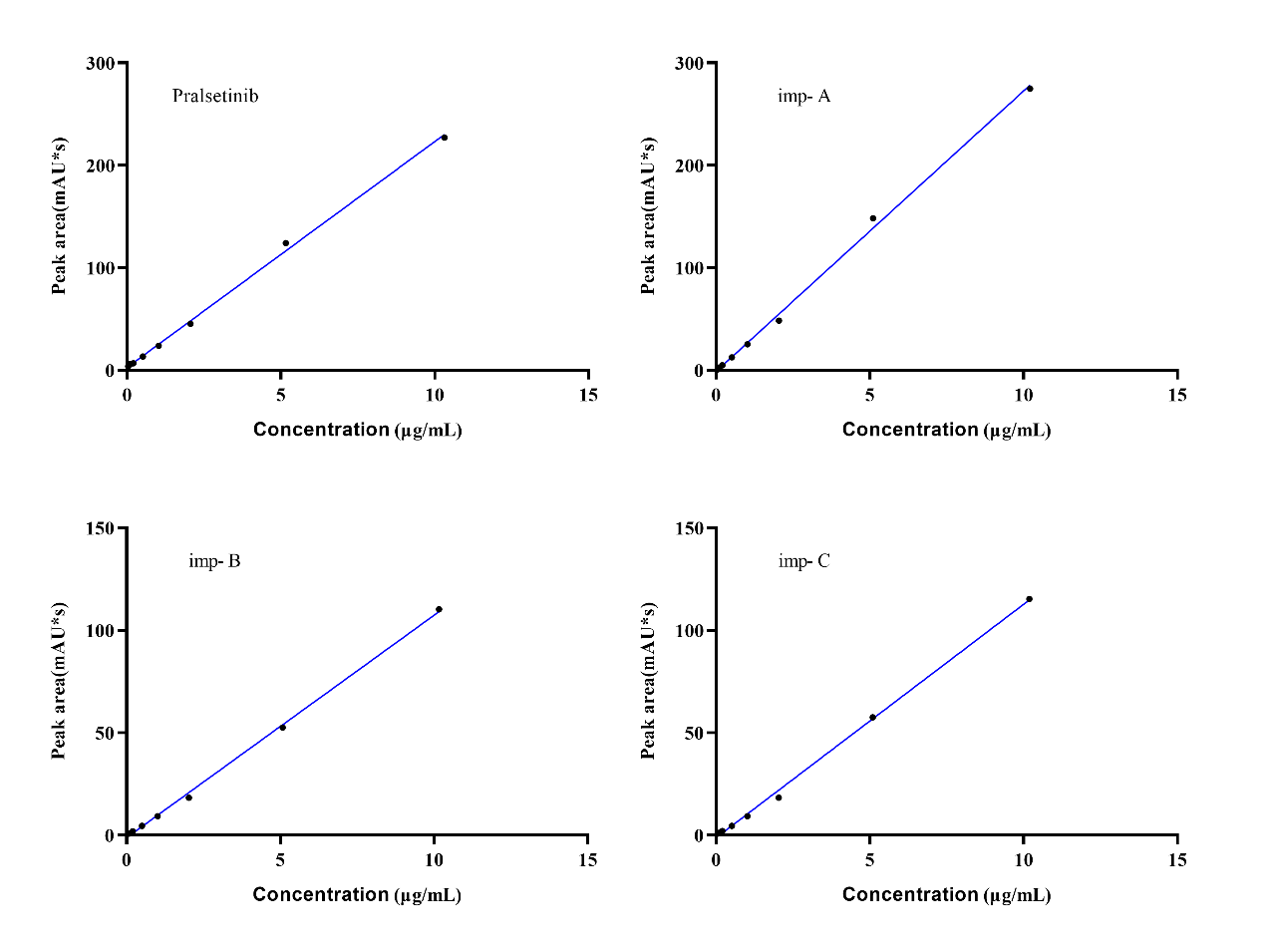


**Figure S2** Regression curves of pralsetinib and related substances

**Table S1** Results of separation tests for pralsetinib and related substances

| **Peak** | **Name** | **Retention time (min)** | **Relative retention time** | **Resolution** |
| --- | --- | --- | --- | --- |
| 1 | imp-A | 9.456 | 0.44 | – |
| 2 | imp-B | 11.242 | 0.53 | 9.56 |
| 4 | imp-C | 13.719 | 0.64 | 15.05 |
| 5 | Pralsetinib | 21.300 | 1.00 | 41.98 |

**Table S2** Results of forced degradation tests

| **Forced Degradation Condition** | **Number of impurities（>0.03 %）** | **Content of main peak (%)** | **Minimum resolution between main peak and impurities** | **Minimum resolution among impurities** | **Equilibrium (%)** |
| --- | --- | --- | --- | --- | --- |
| Undegradation | 3 | 99.61 | 13.18 | 17.60 | 100 |
| Acid degradation | 8 | 95.81 | 4.65 | 2.36 | 96.8 |
| Base degradation | 3 | 98.57 | 14.60 | 45.34 | 98.7 |
| Oxidative degradation | 11 | 94.53 | 4.81 | 1.78 | 95.9 |
| Heat degradation | 3 | 99.68 | 7.82 | 62.91 | 101.5 |
| Photolytic degradation | 5 | 99.05 | 15.11 | 5.45 | 99.2 |

**Table S3** Test results of pralsetinib sample solution stability

| **Time/h** | **Number of impurities** | **Content of maximum single impurity (%)** | **Content of total impurities (%)** |
| --- | --- | --- | --- |
| 0 | 3 | 0.24 | 0.39 |
| 1 | 3 | 0.25 | 0.41 |
| 2 | 3 | 0.24 | 0.39 |
| 4 | 3 | 0.26 | 0.42 |
| 6 | 3 | 0.25 | 0.41 |
| 8 | 3 | 0.24 | 0.40 |
| 12 | 3 | 0.26 | 0.42 |
| 24 | 3 | 0.27 | 0.45 |
